# Opal: an implementation science tool for machine learning clinical decision support in anesthesia

**DOI:** 10.1007/s10877-021-00774-1

**Published:** 2021-11-27

**Authors:** Andrew Bishara, Andrew Wong, Linshanshan Wang, Manu Chopra, Wudi Fan, Alan Lin, Nicholas Fong, Aditya Palacharla, Jon Spinner, Rachelle Armstrong, Mark J. Pletcher, Dmytro Lituiev, Dexter Hadley, Atul Butte

**Affiliations:** 1 Department of Anesthesia and Perioperative Care, University of California San Francisco, San Francisco, 550 16th St., San Francisco, CA 94158 USA; 2 Bakar Computational Health Sciences Institute, University of California San Francisco, San Francisco, CA USA; 3 School of Medicine, University of California San Francisco, San Francisco, CA USA; 4 Undergraduate Studies, University of California Berkeley, Berkeley, CA USA; 5 Department of Cellular and Molecular Pharmacology, University of California San Francisco, San Francisco, CA USA; 6 Department of Epidemiology and Biostatistics, University of California San Francisco, San Francisco, CA USA

**Keywords:** Implementation science, Anesthesia information management system (AIMS), Machine learning, Artificial intelligence, Data organization and processing, Medical outcome monitoring and prediction

## Abstract

Opal is the first published example of a full-stack platform infrastructure for an implementation science designed for ML in anesthesia that solves the problem of leveraging ML for clinical decision support. Users interact with a secure online Opal web application to select a desired operating room (OR) case cohort for data extraction, visualize datasets with built-in graphing techniques, and run in-client ML or extract data for external use. Opal was used to obtain data from 29,004 unique OR cases from a single academic institution for pre-operative prediction of post-operative acute kidney injury (AKI) based on creatinine KDIGO criteria using predictors which included pre-operative demographic, past medical history, medications, and flowsheet information. To demonstrate utility with unsupervised learning, Opal was also used to extract intra-operative flowsheet data from 2995 unique OR cases and patients were clustered using PCA analysis and k-means clustering. A gradient boosting machine model was developed using an 80/20 train to test ratio and yielded an area under the receiver operating curve (ROC-AUC) of 0.85 with 95% CI [0.80–0.90]. At the default probability decision threshold of 0.5, the model sensitivity was 0.9 and the specificity was 0.8. K-means clustering was performed to partition the cases into two clusters and for hypothesis generation of potential groups of outcomes related to intraoperative vitals. Opal’s design has created streamlined ML functionality for researchers and clinicians in the perioperative setting and opens the door for many future clinical applications, including data mining, clinical simulation, high-frequency prediction, and quality improvement.

**Supplementary Information:**

The online version of this article contains supplementary material available 10.1007/s10877-021-00774-1.

## Introduction

The application of machine learning (ML) algorithms toward clinical decision support (CDS) has been demonstrated to be effective in many fields of medicine [1, 2]. Within clinical anesthesia, ML models have been trained to predict numerous clinical outcomes including intraoperative hypotension [3], post-operative length to discharge [4], and post-operative mortality [5, 6]. However, there remains a significant disparity between the rate of development of ML models and their clinical integration within the perioperative setting.

Clinical dashboards are the primary approach to data management within the perioperative environment [7, 8]. One example is the anesthesia information management system (AIMS), a comprehensive system of hardware and software integrated with the electronic health record (EHR) that combines perioperative documentation review with the intraoperative record [9, 10]. AIMS allow for a streamlined provider workflow with improved perioperative assessments, automated clinical decision support, quality improvement measures, and billing [10–13]. A survey of academic medical institutions found that 75% of U.S. academic anesthesiology departments had adopted AIMS in 2014, with 84% expected to do so by 2018–2020 [14].

Due to its broad national adoption, AIMS has been widely utilized for CDS [15–17]. AIMS-based systems have been implemented to target post-operative nausea and vomiting [18], gaps in blood pressure monitoring [19], intraoperative hypotension and hypertension [20], hypoxia and acute lung injury [21], and quality and billing improvement measures [22–24]. High-frequency data updating AIMS-based systems have also been developed including Smart Anesthesia Manager (SAM), a near real-time AIMS-based system for addressing issues in clinical care, billing, compliance, and material waste [25]. However, SAM and other AIMS-based systems have not yet been shown to be compatible with ML algorithms.

ML has the potential to significantly reshape the intraoperative course of care. Wijnberge et al. demonstrated that an ML-based early warning system reduced median time of intraoperative hypotension [26]. However, prediction of hypotension in this study was performed solely based on the intraoperative arterial waveform without additional data from the EHR. While a single-variable ML predictor has clinical value, we believe that a multi-variable ML system that combines intraoperative and EHR data can broadly improve effectiveness of anesthesia care.

Here we discuss Opal, a specialized AIMS-based ML system designed for clinical and research operations that serves as a seamless connection between the EHR and health care providers. Opal provides expedient data extraction, adjustable queries by provider-determined cohort selection, and a detailed dashboard for comprehensive data visualization and implementation of ML algorithms. This comprehensive approach to clinical ML provides a unified solution to the traditional problems of data accessibility, provider usability, and security.

As a demonstration of Opal’s capabilities, we have developed two simple machine learning models. One supervised learning model that predicts post-operative acute kidney injury (AKI) and a clustering model that uses intra-operative flowsheet values to cluster patients based on intraoperative vitals. Post-operative AKI is an important outcome to predict because AKI is associated with dangerous cardiac events and increased mortality. If early warning is available for an anesthesiologist, there are interventions available to reduce the likelihood that patient will have a poor outcome. Here we provide the development of these models and a simple internal validation of the AKI model, but external validation of both models would be recommended before use.

## Methods

**Fig. 1:**
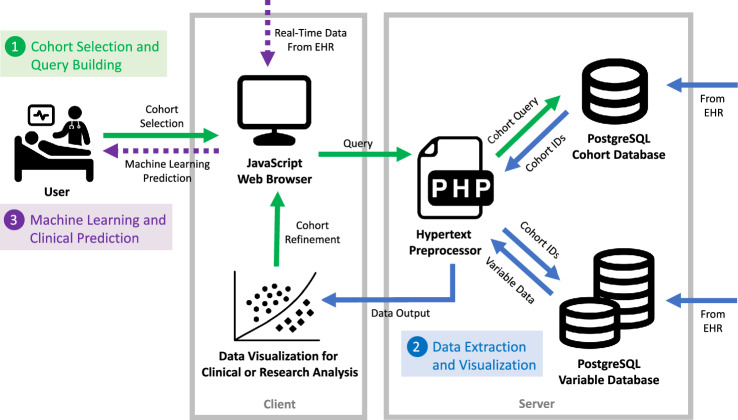
Overview of the Opal dataflow structure. The dataflow of Opal is outlined in three phases: cohort selection, data extraction and visualization, and machine learning prediction. A cohort is first specified by the user to build a query for the Opal database. Data is then extracted via a two-step process with a superficial query of the cohort database to identify appropriate case IDs followed by a detailed query of the variable database to extract data from those cases for output. Once data has been extracted to the client, the user has the opportunity to visualize the data on the Opal dashboard, refine the cohort to better match the desired specifications, or export the data to an external platform for model training or any other research application. If machine learning prediction is desired the user can upload model parameters back into the Opal client, which can then use real-time data asynchronously from the EHR to generate live predictions. Icons used in generating this diagram were obtained from the Noun Project and are cited in the article references. *EHR* electronic health record, *ID* identifier

Data retrieval was approved by the UCSF institutional review board (IRB #17–23,204) from UCSF’s EHR data warehouse for all operative cases from 2012 onward and the requirement for informed consent was waived by the IRB. Opal is an online application for physician use that performs streamlined ML for prediction and classification purposes within the clinical setting. It consists of a JavaScript web client and a PostgreSQL database that is populated with data from the EHR. Users interact with the web client as a front-end interface to extract information from the database based on a selected cohort. An overview of the Opal dataflow is provided in Fig. 1 and is divided into three key phases: cohort selection, data extraction and visualization, and clinical prediction.

### Cohort selection and query building

**Fig. 2:**
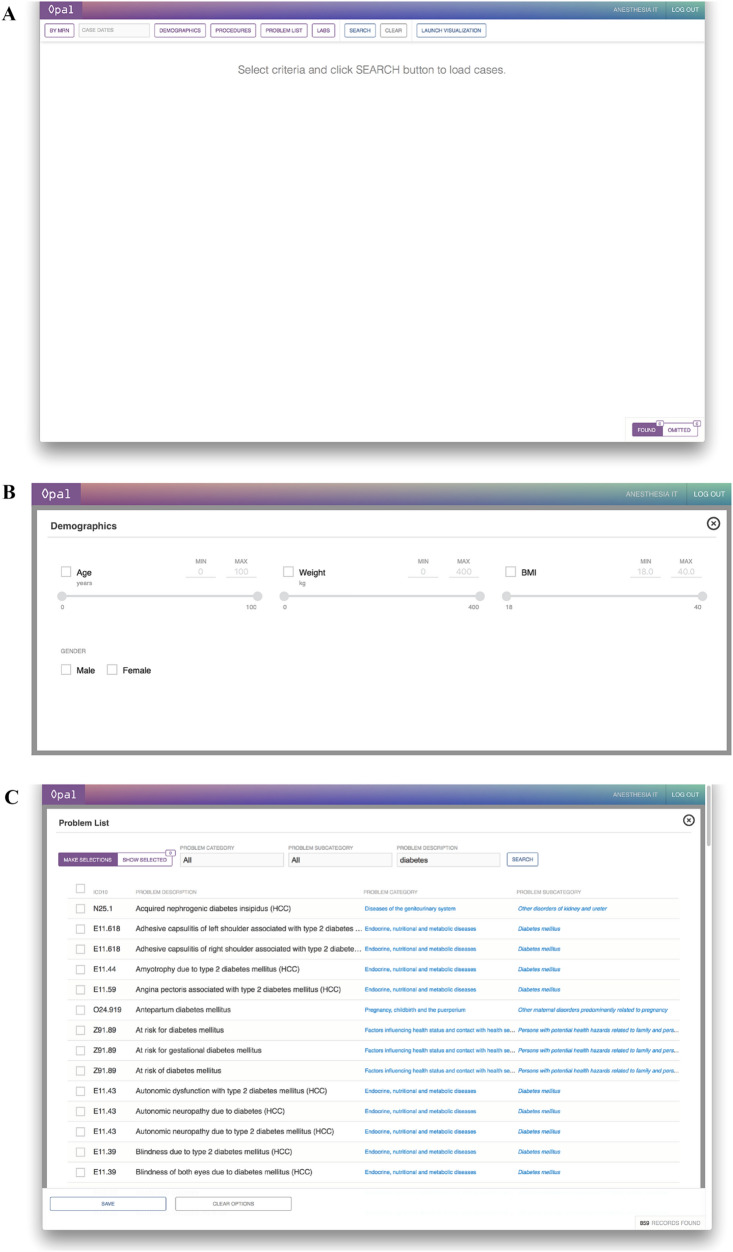

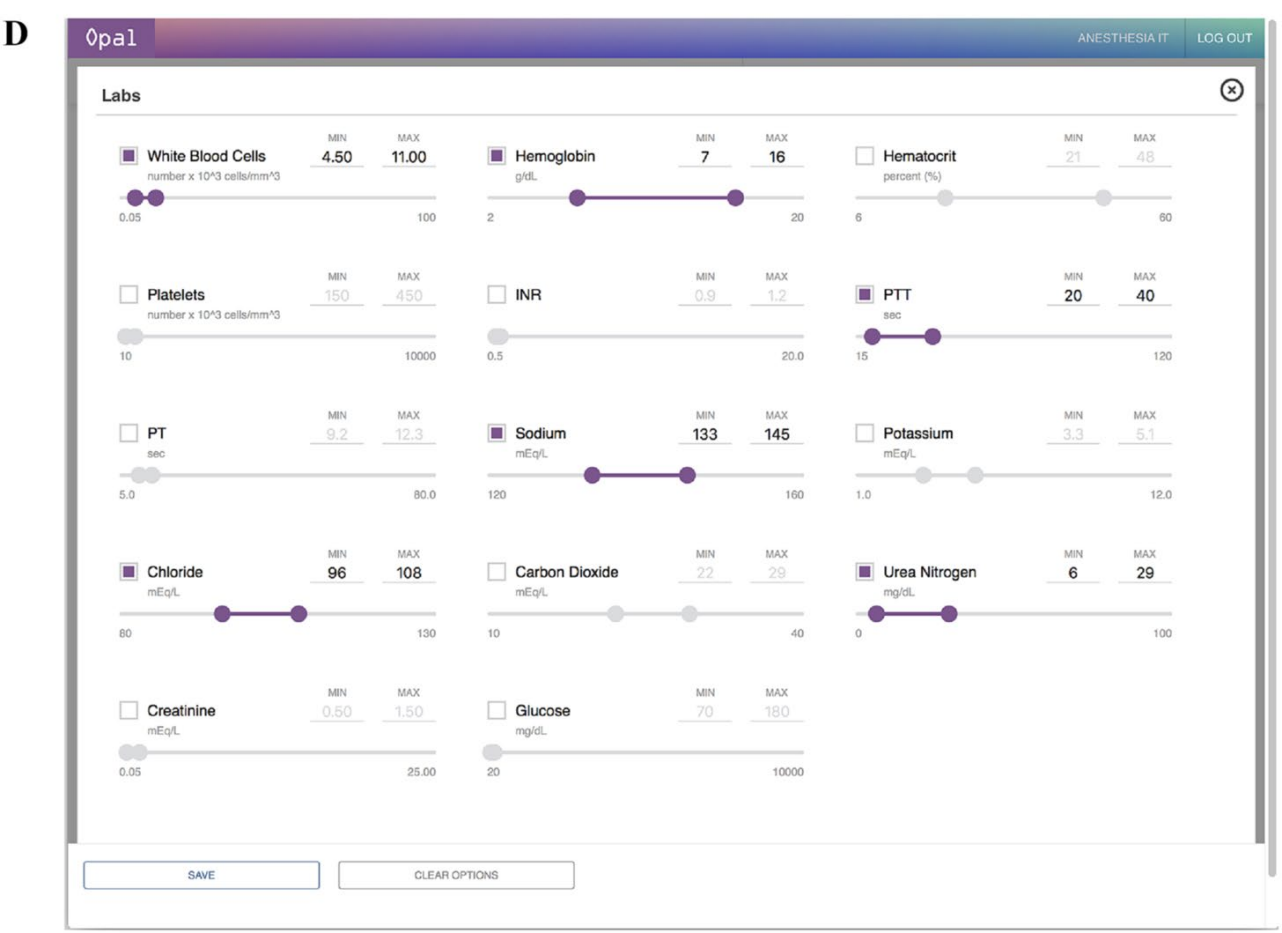
Opal web dashboard for dynamic cohort selection. The Opal web dashboard can be accessed through any in-system web browser and is used for cohort selection to generate the desired dataset. Desired case characteristics are selected on the dashboard interface through the use of sliding scales for quantitative variables and checkbox selections for qualitative variables. **A** Opal dashboard landing page. **B** Selection interface for demographics. **C** Selection interface for problem list. **D** Selection interface for laboratory values

During dynamic cohort selection, the user interacts with a client dashboard on the web browser that allows for selection of retrospective cases by patient identifier, time period, patient demographics, procedures, problem lists, and pre-operative laboratory values (Fig. 2). Prior to data visualization, users are provided with a sample size estimate for their given set of parameters, which may be re-adjusted to match the desired sample size prior to submission. The user is also required to indicate a post-operative outcome of interest from a list of options, with examples including all-cause mortality, delirium, acute kidney injury, and nausea and vomiting. Once selection criteria are finalized, a dynamic SQL query of the variable database is executed when the user selects “Launch Visualization” on the dashboard.

### Data extraction and visualization

**Fig. 3:**
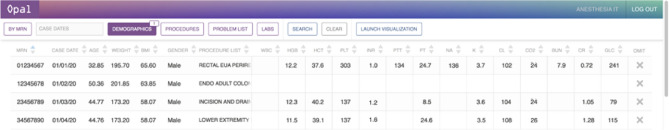
Sample table of resulting cases from cohort selection. Several rows from a sample table of cases are displayed here to serve as an example of the list of cases which is returned to the user on the Opal web client following cohort selection and initial data extraction. Each row represents a separate case, with the corresponding case identifier, case date, and clinical data listed for each case. From this screen, users may conduct case review on individual cases, choose to omit individual cases from the cohort by clicking on the rightmost “Omit” column, or launch visualization in the toolbar located at the top of the screen. All case data provided in this figure are falsified and serve only as a viewing example. *BMI* body mass index, *BUN* blood urea nitrogen, *Cl* Chloride, *CO2* carbon dioxide, *CR* creatinine, *GLC* glucose, *HGB* hemoglobin, *INR* international normalized ratio, *K* potassium, *MRN* medical record number, *NA* sodium, *PLT* platelets, *PT* prothrombin time, *PTT* partial thromboplastin time, *WBC* white blood count

There are currently 29,004 unique case IDs available for extraction within the Opal database that correspond to operative cases within the University of California, San Francisco health system between December 7, 2016 and December 31, 2019. The Opal database serves as a PostgreSQL database that is structurally divided into two separate partitions: a smaller cohort database that stores a list of case identifiers (ID) with corresponding clinical features that correlate with cohort selection, and a larger feature database that stores the complete set of medical features by case ID for data retrieval. Both databases will be updated weekly from the EHR and stored separate from the EHR, which allows for ML-optimized data processing. Large structural changes to the data are performed in this step (e.g. joining of medications with multiple names, validation of lab ranges, calculation of oral morphine equivalents). Once a cohort has been finalized, data is extracted from the variable database and outputted to the JavaScript web client for review and visualization (Fig. 3). For large datasets, the web client can be bypassed and the data can be exported directly to an external source for large-scale analysis.

When data is first passed into the JavaScript web client, a second step of automated data processing occurs to maximize data accuracy and completeness (see supplement for more details). Further data cleaning steps that were otherwise not performed in the PostgreSQL database occur here (e.g. regression imputation of missing values, merging of duplicate values, separation of boluses and infusions). Users have the option to omit this step if they prefer manual processing, but automated pre-processing occurs at default.

Users may access the Opal web client from any secure, in-network workstation including verified desktops, laptops, and mobile devices. The web client interface allows for users to review individual cases within the cohort. In the case review format, users can view vital signs, fluid administration, laboratory values, medications, and ventilation of retrospective cases in a chronologically ordered fashion. This is further discussed in the results section below. Opal also supports in-client ML though both unsupervised (K-means clustering) and supervised (logistic regression, random forest, gradient boosting machines) architectures, which can be used for comparison of current patient with retrospective cases. Deletion or omission of individual cases can also be performed at this time for further data processing. Once the user finalizes the cohort and meets the appropriate necessary IRB and other data safety requirements, the cases can be exported to an external platform via a JavaScript object notation (JSON) or comma-separated value (CSV) file for external analysis and model training. The case data can then be utilized for independent research or used to train a machine learning model to integrate back into Opal.

### Machine learning and clinical prediction

Opal can be utilized for clinical ML prediction. In its current iteration, Opal supports logistic regression (LR), random forest (RF), and gradient boosting machines (GBM) architectures, with support for additional architectures, such as neural networks. In order to perform clinical prediction in Opal, users can either first train a ML model on an external platform and then upload the model parameters back within Opal or train a smaller dataset using the Opal platform. For example, in order to employ a LR architecture users can provide an outcome of interest, a list of predictive features, and their corresponding weights. Once the user has defined the model within Opal, high-frequency data updates for a prospective patient can be retrieved by the JavaScript client from the EHR API to perform prediction on prospective cases. Models can be used for single cases to answer clinical questions, for batch prediction on a set of multiple cases, or can saved to be used for future use such as prospective analysis of predictive value for research models. All model prediction is performed within the JavaScript web browser, thereby increasing accessibility and usability for Opal users.

### Data security

Security remains a large issue for all EHR and AIMS-based data systems, and Opal is designed to maximize security at each step of the data transfer. Since the Opal web client is available via web browser, it may be securely accessed on any encrypted, in-network device. A valid dual-authentication user sign-on in addition to pre-approved device encryption are baseline requirements for accessing Opal. The subnet for the web client is private. The PostgreSQL databases are stored on secure, encrypted servers and no data is directly stored on the device at any time prior to a data export request from the user. As with most EHRs, logs are kept on every user and instance that accesses data on Opal for use tracking, and auditing is performed on an external server. Penetration testing is performed on a regular basis to ensure system security.

### Example models developed with Opal

By providing streamlined access to EHR data, Opal allows for a variety of direct data analysis applications. Here we provide two discrete examples of data extraction through Opal, for use in ML analysis of acute kidney injury (AKI) and intraoperative vitals clustere analysis. Supervised learning via a gradient boosting machine (GBM) was conducted to train a model for the prediction of prospective AKI patients, while unsupervised learning via K-means clustering was used to analyze intraoperative vitals for hypothesis generation.

### Gradient boosting machine for prediction of post-operative acute kidney injury

After above-mentioned IRB was attained, a cohort of 29,004 adult operative cases at UCSF hospitals Moffitt-Long and Mission Bay between December 7, 2016 and December 31, 2019 available in the Opal database were extracted via the Opal pipeline. The patient characteristics from the cohort are outlined in Table 1. A binary stage 1 or greater AKI outcome was defined using the KDIGO criteria [27] of a post-operative creatinine increase of 0.3 mg/dL or greater (chosen over AKIN and RIFLE criteria) [28]. Of the 29,004 cases, patients without a pre-operative creatinine value were excluded leaving 8,858 cases. Post-operative AKI was predicted pre-operatively at the moment immediately prior to transporting the patient to the operating room for anesthesia. 155 clinical variables were extracted for all cases, including patient demographics, medications, ICD10 codes, laboratory values, surgery-specific risks, and vital signs. Data pre-processing including standardizing, imputation, dataset merging, and visualization served to validate data quality. Sample size was chosen based upon the maximum available data with available outcomes to optimize training of the model. Missing data in input variables were imputed to zero in some variables such as medication administrations and ICD10 codes, but in other cases were not imputed and left as NaN values as the missing value provides added predictive value in the model we chose (XGBoost). 74 categorical variables were one-hot-encoded and ICD10 codes were enumerated by category for each patient. Variables that contained information after the prediction timepoint were truncated to the end of the anesthetic case. The 8,858 cases were split into training (80%) and test (20%) datasets. Because of the class imbalance and in order to improve the model sensitivity, AKI cases were oversampled in the training set to match the number of non-AKI cases. We compared this model to a reference logistic regression with a similar training/test split, using the most important variables identified in the gradient boosting model using the Shapley method of machine learning interpretation.

**Table 1:** Cohort characteristics for prediction of acute kidney injury

	No AKI	AKI	P*
Total cases	8474 (95.7)	384 (4.3)	
Age (years)	60.1 (15.9)	58.7 (14.6)	0.08
Gender			
Female	3035 (45.7)	132 (39.5)	0.03
Male	3603 (54.3)	202 (60.5)	
Body mass index (kg/m^2^)	27.7 (7.7)	28.1 (7.9)	0.29
Weight (kg)	79.2 (23.0)	81.4 (25.9)	0.13
ASA class			
1	105 (1.6)	0 (0.0)	< 0.001
2	1619 (24.7)	31 (9.5)	
3	4209 (64.2)	244 (74.8)	
4	621 (9.5)	51 (15.6)	
5	7 (0.1)	0 (0.0)	
ASA E			
No	4165 (62.7)	226 (67.7)	0.079
Yes	2473 (37.3)	108 (32.3)	
Primary service			
Anesthesia	18 (0.3)	1 (0.3)	< 0.001
Breast	5 (0.1)	1 (0.3)	
Cardiac surgery	123 (1.9)	3 (0.9)	
Cardiology	465 (7.0)	22 (6.6)	
Cardiology peds	7 (0.1)		
Gastroenterology	106 (1.6)	7 (2.1)	
General surgery	1184 (17.8)	29 (8.7)	
Genito urology	237 (3.6)	21 (6.3)	
Genito urology peds	1 (0.0)	0 (0.0)	
Gynecology	25 (0.4)	2 (0.6)	
Gynecology oncology	7 (0.1)	0 (0.0)	
Neurological surgery	1038 (15.6)	15 (4.5)	
Ophthalmology	19 (0.3)	2 (0.6)	
Oral Maxillo-facial surgery	66 (1.0)	2 (0.6)	
Orthopedics surgery	1144 (17.2)	29 (8.7)	
Otolaryngology	136 (2.0)	4 (1.2)	
Plastic surgery	269 (4.1)	23 (6.9)	
Pulmonary	103 (1.6)	2 (0.6)	
Thoracic surgery	110 (1.7)	5 (1.5)	
Transplant	809 (12.2)	103 (30.8)	
Vascular surgery	766 (11.5)	63 (18.9)	
30 Day prior admission			
No	3826 (57.6)	201 (60.2)	0.39
Yes	2812 (42.4)	133 (39.8)	
90 Day prior admission			
No	2526 (38.1)	130 (38.9)	0.79
Yes	4112 (61.9)	204 (61.1)	
Case length (h)	179.1 (113.7)	171.6 (108.7)	0.22
Number of allergies	5.9 (8.7)	7.2 (9.6)	0.07
3 Year prior anesthesia cases	2.3 (3.5)	2.6 (3.4)	0.07

Continuous variables are summarized by mean (SD) and categorical variables are summarized by n (%)

*AKI* acute kidney injury, *Peds* pediatrics, *ASA* American Society of Anesthesiologists, *ASA E* emergency surgery, *h* hour, *kg* kilogram, *m* meter

A gradient boosting machine learning decision tree (XGBoost python package) was trained externally to Opal due to the size of the dataset (as mentioned above, these weights can be uploaded to Opal for prediction of new cases). Feature importance was calculated by randomly permuting each variable in the training set and measuring the effect on prediction.

### K-means clustering of intraoperative vitals

The Opal dataflow was used to retrieve data from 2995 unique case IDs corresponding to a continuous period between January 1, 2017 and February 28, 2018. These operative cases were also taken from UCSF where operations occurred at Moffitt-Long hospital and are a subset of the patients described in Table 1. As the training of this model occurred within the Opal infrastructure, we chose a smaller dataset to assure there would be sufficient computational power. A total of 6 variables were included in the analysis, which consisted of intraoperative vital signs. Missing data was imputed with simple forward fill and the remaining missing values were imputed with the value “0”. Time of clustering occurred at the end of the operation.

Data from these case IDs were loaded into the Opal web client. PCA dimension reduction were applied to the input variables and then K-means clustering was performed to partition the cases into two clusters. Case review was performed on individual patients in each cluster to review vital signs for each respective cluster.

## Results

### Gradient boosting machine for prediction of post-operative acute kidney injury

**Fig. 4:**
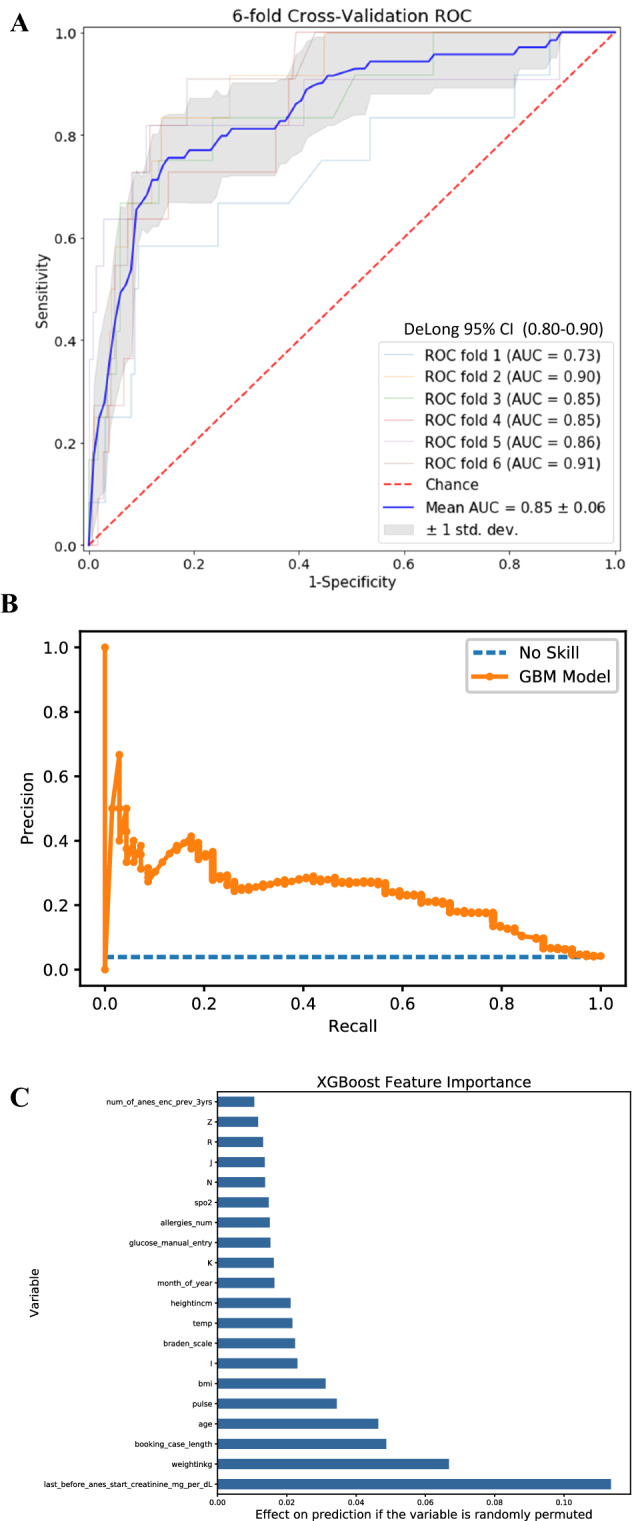
Results from gradient boosting machine for acute kidney injury. 8,858 unique cases with pre-operative creatinine values were extracted from the Opal database and exported to train a gradient boosting machine for the prediction of AKI in post-operative patients. 155 different clinical variables were used, including patient demographics, medications, ICD10 codes, laboratory values, surgery-specific risks, and vital signs. Cases were divided into training (80%) and test (20%) datasets. The model achieved a ROC-AUC of 0.85 [0.80,0.90] when validated on the holdout test set, with a sensitivity of 0.9 and sensitivity of 0.8 at a selected decision threshold of 0.5. The precision-recall curve and a chart listing the most predictive clinical features of the model are provided here as well. Panel C presents the most predictive features in the model in order of importance, with letter variables representing corresponding ICD 10 codes as follows: *I* circulatory system, *K* digestive system, *N* genitourinary system, *J* respiratory system, *R* abnormal lab findings, *Z* factors influencing health status. **A** ROC-AUC curve for the GBM model. **B** Precision-recall curve for the GBM model. **C** List of most important features for the GBM model. *AKI* acute kidney injury, *GBM* gradient boosting machine, *ICD10* International Statistical Classification of Diseases and Related Health Problems, *ROC-AUC* area under the receiver operating curve

Of the 8858 cases, 4.3% of the patients had postoperative AKI based upon the definition described above. Validation of the model on the holdout test dataset yielded an area under the receiver operating curve (ROC-AUC) of 0.85. The 95% confidence interval for the ROC-AUC was 0.80 to 0.90 measured using the DeLong method. At the default probability decision threshold of 0.5, the model sensitivity was 0.9 and the specificity was 0.8. Figure 4 shows the ROC curve and feature importance of the initial retrospective model prediction of AKI. This model performed significantly better than our reference logistic regression model that predicted with a ROC-AUC of 0.73 (0.70–0.76) using the most important variables selected from the gradient boosting model (see SHAP figure in supplemental materials). These results and the details of the reference logistic regression model are shown in the supplement materials.

### K-means clustering of intraoperative vitals

**Fig. 5:**
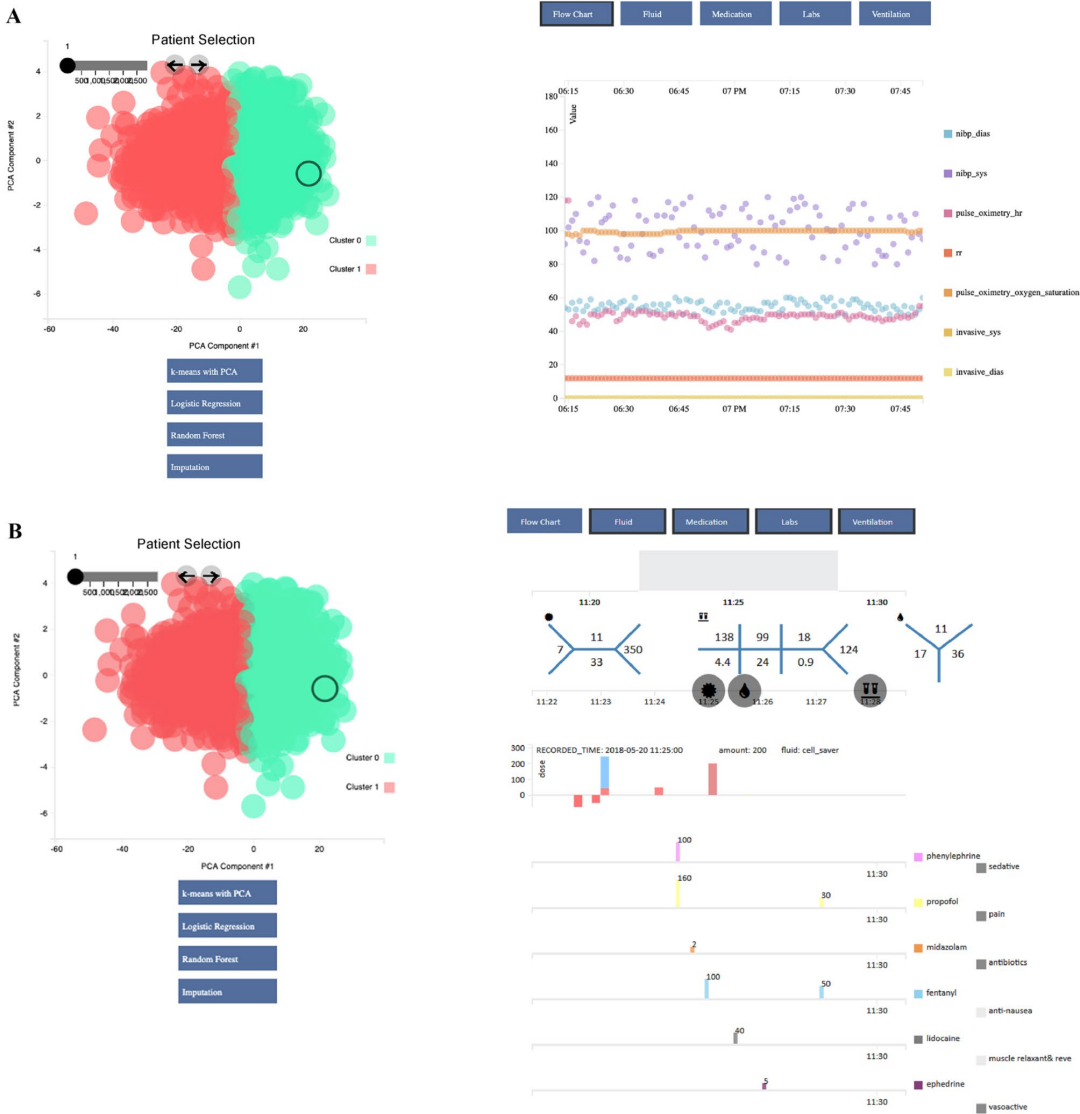
K-means clustering of intraoperative vitals. 2,995 unique cases were extracted from the Opal database and were visualized on the Opal web client for unsupervised machine learning analysis. K-means clustering was performed on the cohort to partition the cases into two clusters. Individual cases were chosen for case review by clicking each circle from the data visualization graph on the left. Case data from the selected case was displayed corresponding to the data categories in the blue toolbar and time frame on the grey timeline selected by the user on the upper right-hand side. Different combinations of the vital signs flowchart, laboratory values, fluids, and medications can be selected at once for viewing. Supervised machine learning architectures including logistic regression and random forest can also be performed by the web application to allow the user to compare a prospective patient with similar past cases. After reviewing the cohort, the user may modify the list of cases to better match his or her research or clinical needs and may export the data to an external platform for further analysis. **A** Individual case analysis of vital signs flow chart. **B** Individual case analysis of laboratory values, fluid administration, and medications. *dbp* diastolic blood pressure, *h* heart rate, *PCA* principal component analysis, *po* pulse oximetry, *rr* respiratory rate, *sbp* systolic blood pressure. The numbers separated by the blue lines in the top right of the image are the laboratory values of the patient showing the Complete Blood Count (CBC), Chemistry 7 (CHEM 7), and coagulation (COAG) in the traditional “fishbone” shorthand representations of these laboratories regularly used in United States medical centers

2995 cases were analyzed using the clustering analysis. Figure 5 demonstrates the results of the K-means clustering after PCA dimension reduction and case review on the Opal dashboard. Opal was able to successfully partition the cases into two distinct groups based on the provided predictive features, thus allowing for prospective clustering of future cases. Performance evaluation was assessed via visual inspection as the goal was hypothesis generation for future investigation.

## Discussion

In this study we present Opal, a comprehensive AIMS-based ML system that designed specifically for large-scale ML. Opal addresses problems of data accessibility, provider usability, and security that have historically limited ML development in medicine.

The greatest strength of the Opal system is its ability to extract large-scale datasets for both research and clinical applications. The EHR is the most widely used data source for training of ML models. Studies that utilize data from the EHR often require manual data extraction, a process which can be both difficult and time-consuming, particularly for large-scale queries. Opal creates a streamlined pipeline for data extraction that is standardized, replicable, and comprehensible. Users may extract data simply by selecting ranges in case criteria without the need for advanced query functions or knowledge of database-specific languages, such as SQL or CACHE. A wide set of set of features are available in Opal including vital signs, laboratory values, problem lists, and procedures which maintains the ability to leverage a large set of features to draw complex associations, one of the fundamental strengths of ML algorithms. Data extracted from Opal is automatically pre-processed with the use of regression imputation, joining of duplicate values and features, and validation of data with exclusion of significant outliers. This greatly lowers the threshold for whom ML can be performed. Opal’s infrastructure also brings us as a medical field much closer to being able to run algorithms that use EHR data in a real-time way to inform and improve clinical care. Many retrospective ML algorithms have been developed, but unless we can build platforms like Opal that integrate with the EHR and can process complex data in ways the EHR is limited, we will not be able to use these ML algorithms for clinical decision support.

One of the greatest criticisms of current ML algorithms is that the statistical process remains opaque the use, thus creating a “black box” algorithm. While Opal does not solve the fundamental issue of statistical obscurity, it does help to bridge the gap between provider and algorithm through the use of dynamic cohort selection and data visualization techniques that increase user feedback and data clarity. The immediate visual feedback allows users to adjust case cohorts as necessary to generate an appropriate target dataset and to better understand the distribution of their datasets prior to formal analysis. This greater familiarity with the data enables hypothesis generation by the user and more accurate training of statistical models.

Data taken from Opal can be used for large-scale statistical analyses or randomized clinical trials by clinicians and researchers alike and creates the opportunity for a broad spectrum of clinical applications including data mining, clinical simulation, high-frequency prediction, and quality improvement. Opal has already been shown to be effective for unsupervised ML with relation to intraoperative vitals and supervised learning for AKI. PCA dimension reduction of the vitals provided the optimal separation of cases, suggesting that non-linear representations of hemodynamic control may be associated with meaningful separations between patient outcomes. Further research can be performed to train a ML model to predict predefined outcomes in future patients, and can readily validated through the Opal framework. Furthermore, this same process can be applied to any clinical outcome of interest, thus opening the door for a multitude of large-scale statistical analyses and clinical trials. While more complex model architectures such as artificial neural networks are not available at this time, they can be readily added to the existing pipeline and are currently being implemented.

We acknowledge several limitations with this study. One widely recognized constraint of EHR data revolves around its inaccuracy or missingness based on inconsistency of provider entry for clinical data. While Opal creates a pipeline for expedited data retrieval from the EHR and includes multiple steps for data processing, it cannot guarantee data accuracy or avoid missingness of EHR data any more that traditional methods of data extraction. Thus, user post-processing of data may still be required for larger datasets to ensure precision of data. Opal does offer several points for data processing, including an automated pre-processing steps in both the PostgreSQL database and the JavaScript web client that includes variable standardization, flagging of abnormal values, and baseline regression imputation for missing values. Despite these steps, we still recognize that data extracted via Opal may still have deficiencies and may require additional review prior to analysis.

One possible unintended consequence of increasing availability of data extraction and ML through Opal is that some users may not have formal statistical training or be as familiar with ML techniques. Therefore, there is some risk of provider misinterpretation of results when using Opal. To counteract this, the Opal interface informs all users that results shown are for research and clinical development purposes and all the data presented by Opal indicate data associations and not causal relationships.

Another limitation is the limited generalizability and lack of interoperability of Opal in both its implementation and data extraction. Since Opal is designed specifically to match the system of our EHR, other institutions may have a difficult time replicating Opal if their EHR system differs greatly in accessibility, structure, and security. Furthermore, data extracted via Opal is limited to a single institution which limits the power and generalizability of clinical trials or analyses that may be generated from this data. However, extracted data can still be shared through an external process mediated by the user. Despite these limitations, we believe that there remains a significant importance in reporting the success of Opal at a single institution to promote the creation of additional EHR data pipelines broadly across the nation to promote ML.

## Supplementary Information

Below is the link to the electronic supplementary material.

Electronic supplementary material 1 (DOCX 520 KB)

## Data Availability

Data is protected health information and cannot be included in submission or made public.

## Code availability

Code is available upon request. Please email Andrew Bishara with requests.

## Declarations

### Research involving animals

Not applicable for this research.

## References

[1] Obermeyer Z, Emanuel EJ (2016). Predicting the future—big data, machine learning, and clinical medicine. N Engl J Med.

[2] Rajkomar A, Oren E, Chen K (2018). Scalable and accurate deep learning with electronic health records. NPJ Digit Med.

[3] Hatib F, Jian Z, Buddi S (2018). Machine-learning algorithm to predict hypotension based on high-fidelity arterial pressure waveform analysis. Anesthesiology.

[4] Safavi KC, Khaniyev T, Copenhaver M (2019). Development and validation of a machine learning model to aid discharge processes for inpatient surgical care. JAMA Netw Open.

[5] Lee CK, Hofer I, Gabel E, Baldi P, Cannesson M (2018). Development and validation of a deep neural network model for prediction of postoperative in-hospital mortality. Anesthesiology.

[6] Hill BL, Brown R, Gabel E (2019). An automated machine learning-based model predicts postoperative mortality using readily-extractable preoperative electronic health record data. Br J Anaesth.

[7] Park KW, Smaltz D, McFadden D, Souba W (2010). The operating room dashboard. J Surg Res.

[8] Franklin A, Gantela S, Shifarraw S (2017). Dashboard visualizations: Supporting real-time throughput decision-making. J Biomed Inform.

[9] Stonemetz J (2011). Anesthesia information management systems marketplace and current vendors. Anesthesiol Clin..

[10] Shah NJ, Tremper KK, Kheterpal S (2011). Anatomy of an anesthesia information management system. Anesthesiol Clin.

[11] Simpao AF, Rehman MA (2018). Anesthesia information management systems. Anesth Analg.

[12] O’Sullivan CT, Dexter F, Lubarsky DA, Vigoda MM (2007). Evidence-based management assessment of return on investment from anesthesia information management systems. AANA J.

[13] Ehrenfeld JM, Rehman MA (2011). Anesthesia information management systems: a review of functionality and installation considerations. J Clin Monit Comput.

[14] Stol IS, Ehrenfeld JM, Epstein RH (2014). Technology diffusion of anesthesia information management systems into Academic Anesthesia Departments in the United States. Anesth Analg.

[15] Nair BG, Gabel E, Hofer I, Schwid HA, Cannesson M (2017). Intraoperative clinical decision support for anesthesia. Anesth Analg.

[16] Simpao AF, Tan JM, Lingappan AM, Gálvez JA, Morgan SE, Krall MA (2017). A systematic review of near real-time and point-of-care clinical decision support in anesthesia information management systems. J Clin Monit Comput.

[17] Chau A, Ehrenfeld JM (2011). Using real-time clinical decision support to improve performance on perioperative quality and process measures. Anesthesiol Clin.

[18] Kooij FO, Klok T, Hollmann MW, Kal JE (2008). Decision support increases guideline adherence for prescribing postoperative nausea and vomiting prophylaxis. Anesth Analg.

[19] Ehrenfeld JM, Epstein RH, Bader S, Kheterpal S, Sandberg WS (2011). Automatic notifications mediated by anesthesia information management systems reduce the frequency of prolonged gaps in blood pressure documentation. Anesth Analg.

[20] Nair BG, Horibe M, Newman S-F, Wu W-Y, Peterson GN, Schwid HA (2014). Anesthesia information management system-based near real-time decision support to manage intraoperative hypotension and hypertension. Anesth Analg.

[21] Kheterpal S, Gupta R, Blum JM, Tremper KK, O’Reilly M, Kazanjian PE (2007). Electronic reminders improve procedure documentation compliance and professional fee reimbursement. Anesth Analg.

[22] Blum JM, Stentz MJ, Maile MD (2013). Automated alerting and recommendations for the management of patients with preexisting hypoxia and potential acute lung injury: a pilot study. Anesthesiology.

[23] Spring SF, Sandberg WS, Anupama S, Walsh JL, Driscoll WD, Raines DE (2007). Automated documentation error detection and notification improves anesthesia billing performance. Anesthesiology.

[24] Freundlich RE, Barnet CS, Mathis MR, Shanks AM, Tremper KK, Kheterpal S (2013). A randomized trial of automated electronic alerts demonstrating improved reimbursable anesthesia time documentation. J Clin Anesth.

[25] Nair BG, Newman S-F, Peterson GN, Schwid HA (2013). Smart anesthesia manager (SAM)—a real-time decision support system for anesthesia care during surgery. IEEE Trans Biomed Eng.

[26] Wijnberge M, Geerts BF, Hol L (2020). Effect of a machine learning-derived early warning system for intraoperative hypotension vs standard care on depth and duration of intraoperative hypotension during elective noncardiac surgery. JAMA.

[27] Kellum JA, Lameire N, Aspelin P (2012). Kidney disease: Improving global outcomes (KDIGO) acute kidney injury work group. KDIGO clinical practice guideline for acute kidney injury. Kidney Int Suppl..

[28] Luo X, Jiang L, Du B, et al. A comparison of different diagnostic criteria of acute kidney injury in critically ill patients. 2014;1–8. 10.1186/cc13977 PMC4227114 25005361

[29] Coquet, Adrien. "Sick.” From the Noun Project. Retrieved March 27, 2020.

[30] Lareo, Sebastian Belalcazar. "Monitor.” From the Noun Project. Retrieved March 27, 2020.

[31] Nociconist. "PHP.” From the Noun Project. Retrieved March 27, 2020.

[32] Nociconist. "Database.” From the Noun Project. Retrieved March 27, 2020.

[33] Aiden Icons. "Database.” From the Noun Project. Retrieved March 27, 2020.

[34] Mbarki, Mohamed. "Machine Learning.” From the Noun Project. Retrieved March 27, 2020.

[35] Product Pencil. "Deep Learning.” From the Noun Project. Retrieved March 27, 2020.

