# Supplementary material for: Opal: an implementation science tool for machine learning clinical decision support in anesthesia
Source: J Clin Monit Comput. 2021 Nov 27;36(5):1367–77. doi: 10.1007/s10877-021-00774-1 (PMC9275816; doi:10.1007/s10877-021-00774-1)
Supplement: Supplementary file 1 — Electronic supplementary material 1 (DOCX 520 KB) [file 10877_2021_774_MOESM1_ESM.docx]

**JavaScript packages used in Opal:**

Visualization Tools

D3.js- A powerful tool that has been used for complex visualizations around the world and was featured on the New York Times website (<https://www.nytimes.com/by/mike-bostock>).

D3_shape.js - A component of D3 that allows for varied shapes of graph symbols.

D3_slider.js- A component of D3 that allows for a slider tool to manipulate visualization tools.

tensorflow_vis.js-neural network visualization tools

Machine Learning Tools

ML.js- includes decision tree ML, Logistic regression, unsupervised learning (k-means, PCA)

tensorflow.js- neural network tools

Customized tools built by the Opal team:

Imputation tools

Data preprocessing tools (see below)

ROC graphing tools

Lab fishbone graphing tools

Medication infusion and bolus parsing tools

| Transformations by JavaScript | Transformations by PostgreSQL |
| --- | --- |
| Choosing which variable is the real invasive blood pressure between ART and ABP | Pivoting datasets so that time event is each row and each column has a different variable |
| Imputation of null values in the vitals | Grouping medications by name |
| Which anesthetic gas is being used defined by a different variable for each anesthetic gas | Grouping medications by bolus and by infusion |
| Grouping drugs by general category | Calculation of number of oral morphine equivalents from all the opioids used |
| Grouping laboratory values by CBC, Chemistry, and Coagulation | Parsing data by time to make sure we are gathering data preop data, intraoperative data, postoperative data |
| Converting infusion drug delivery data to minute by minute data | Joining multiple tables |
| Some measurements are not taken minute by minute so data needs to be reformatted to reflect "sparse" events |  |
| Sorting the data by time |  |
| Pivoting some data structures to reorganize for ingestion by machine learning models |  |
| Joining multiple different data sources |  |

Variables included in the AKI model

| **Variables included in acute kidney injury model prediction** | **demographics** | **home medications** | **preoperative variables** |
| --- | --- | --- | --- |
|  | age | medication name | temperature |
|  | gender |  | temperature source |
|  | weight in kilograms |  | pulse |
|  | bmi |  | heart rate source |
|  | height in centimeters |  | respiratory rate |
|  | race |  | blood pressure method of measurement |
|  | ethnicity |  | pulse oximeter oxygent saturation |
|  | number of anesthetic encounters in previous 3 years |  | blood glucose measure point of care |
|  | allergies |  | body surface area measure (mosteller formula) |
|  | number of allergies |  | pain assessment |
|  | number of "severe" reactions to allergies |  | pain level |
|  | past medical history (number of ICD10 codes in each category) |  | acceptable pain level for patient |
|  | marital status |  | pain type |
|  | insurance payor |  | pain location |
|  | how patients prefer to learn new subjects (nursing note) |  | pain character |
|  | patient barriers to learning |  | effect of pain on daily activities |
|  | primary language |  | multiple pain sites? |
|  | interpreter needed |  | current pain interventions being employed for patient |
|  | smoking status |  | thermoregulation interventions |
|  | alcohol use |  | assessment of attention (spell world backwards) |
|  |  |  | illness severity subjective from nurse (mild to severe) |
|  |  |  | AWOL assessment (orientation) |
|  |  |  | access to transportation |
|  |  |  | dressing |
|  |  |  | grooming |
|  |  |  | bathing |
|  |  |  | in and out of bed? |
|  |  |  | feeding? |
|  |  |  | weakness in hands? |
|  |  |  | weakness in legs? |
|  |  |  | bowel bladder habits |
|  |  |  | vision issues |
|  |  |  | hearing right ear |
|  |  |  | hearing left ear |
|  |  |  | mobility issues? |
|  |  |  | elimination? |
|  |  |  | history of falls |
|  |  |  | schmid fall risk score |
|  |  |  | braden score |
|  |  |  | stratify fall risk |
|  |  |  | neuro problem documented? |
|  |  |  | cardiac problem documented? |
|  |  |  | musculoskeletal problem documented? |
|  |  |  | GU problem documented? |
|  |  |  | psychosocial problem documented? |
|  |  |  | violence abuse assessment |
|  |  |  | social work consult needed |
|  |  |  | sucide risk |
|  |  |  | pain or discomfort |
|  |  |  | pain relieving factors |
|  |  |  | unplanned weight loss |
|  |  |  | difficulty chewing |
|  |  |  | difficulty swalling |
|  |  |  | tube feeding? |
|  |  |  | total parental nutrition |
|  |  |  | presence of pressure ulcer |
|  |  |  | nonhealing wound |
|  |  |  | dietician consult needed |
|  |  |  | oxygen device needed |
|  |  |  | braces devices and/or sensory aids |
|  |  |  | sleep habit details |
|  |  |  | glasgow coma scale score |
|  |  |  | arrived from |
|  |  |  | patient in the early recovery after surgery (ERAS) patient |
|  |  |  | booking case length |
|  |  |  | case location |
|  |  |  | 30 day prior admission? |
|  |  |  | 90 day prior admssion? |
|  |  |  | patient admission source |
|  |  |  | operating room team service primary for patient |
|  |  |  | emergency contact listed? |
|  |  |  | American Society of Anesthesiologists (ASA) class |
|  |  |  | American Society of Anesthesiologists (ASA) emergency? |
|  |  |  | case classification |

Supplemental Table 2: Past Medical History ICD10 Categories

| A00-B99 Certain infectious and parasitic diseases |
| --- |
| C00-D49 Neoplasms |
| D50-D89 Diseases of the blood and blood-forming organs and certain disorders involving the immune mechanism |
| E00-E89 Endocrine, nutritional and metabolic diseases |
| F01-F99 Mental, Behavioral and Neurodevelopmental disorders |
| G00-G99 Diseases of the nervous system |
| H00-H59 Diseases of the eye and adnexa |
| H60-H95 Diseases of the ear and mastoid process |
| I00-I99 Diseases of the circulatory system |
| J00-J99 Diseases of the respiratory system |
| K00-K95 Diseases of the digestive system |
| L00-L99 Diseases of the skin and subcutaneous tissue |
| M00-M99 Diseases of the musculoskeletal system and connective tissue |
| N00-N99 Diseases of the genitourinary system |
| O00-O9A Pregnancy, childbirth and the puerperium |
| P00-P96 Certain conditions originating in the perinatal period |
| Q00-Q99 Congenital malformations, deformations and chromosomal abnormalities |
| R00-R99 Symptoms, signs and abnormal clinical and laboratory findings, not elsewhere classified |
| S00-T88 Injury, poisoning and certain other consequences of external causes |
| V00-Y99 External causes of morbidity |
| Z00-Z99 Factors influencing health status and contact with health services |

SHAP is a machine learning “explainer” python package. This shows the top variables and their relationship **within the model** to the outcome of interest: acute kidney injury.


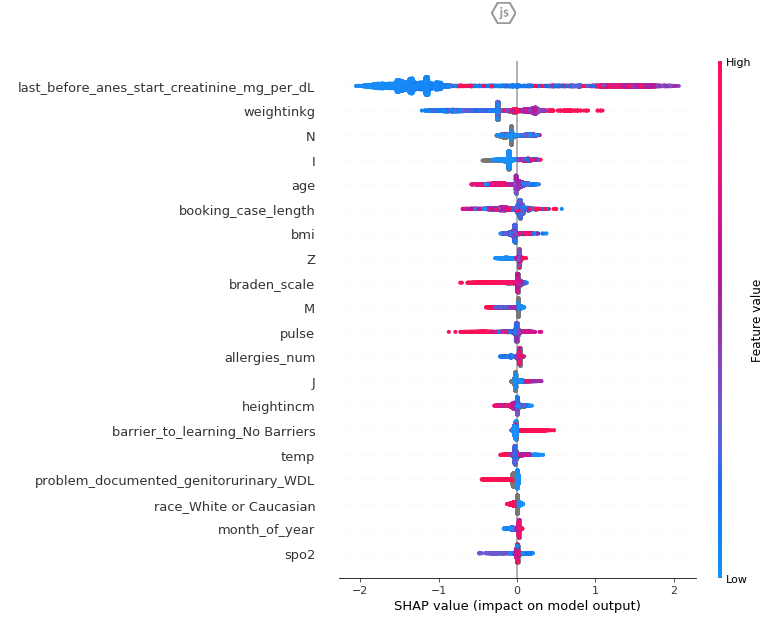


Logistic Regression reference model for predicting acute kidney injury:

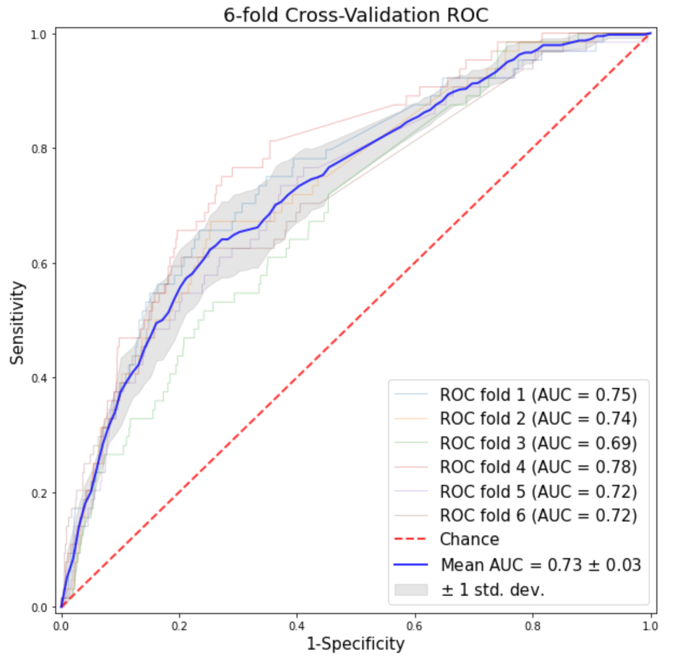


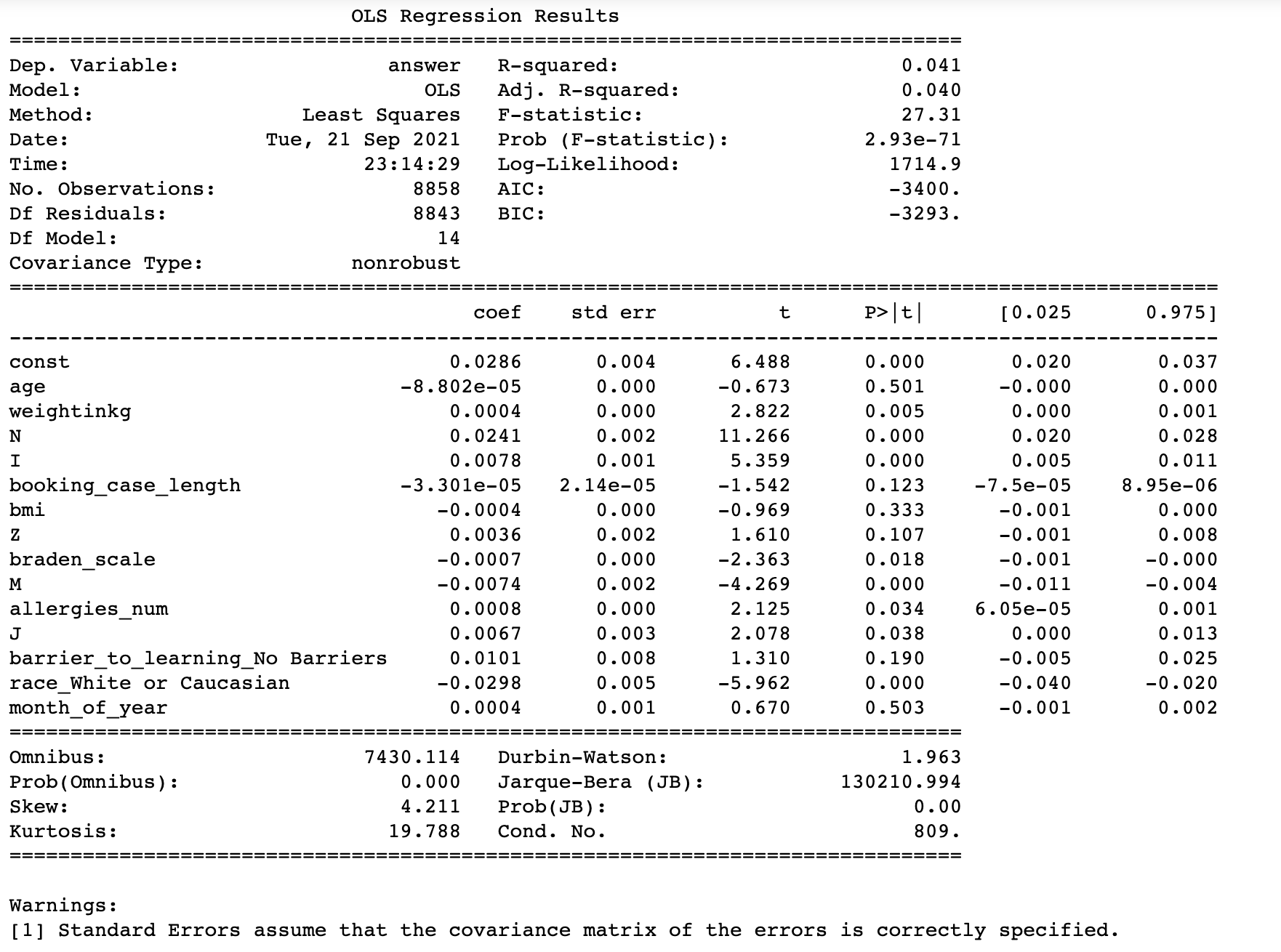


Unsupervised cluster analysis variables:

| non-invasive diastolic blood pressure mean value of all minutes of case |
| --- |
| non-invasive systolic blood pressure mean value of all minutes of case |
| non-invasive mean blood pressure mean value of all minutes of case |
| pulse oximeter heart rate value mean value of all minutes of case |
| respiratory rate mean value of all minutes of case |
| pulse oximetere oxygen saturation mean value of all minutes of case |
